# Mechanical behavior of Port Basin Bulkheads Reinforced with novel multi-anchored sheet piles

**DOI:** 10.1371/journal.pone.0340503

**Published:** 2026-01-08

**Authors:** Jiayun Gao, Xiaojun Li, Yan Zhu, Jie Jiang, Shaojie Tian, Bowen Kang

**Affiliations:** 1 School of Ocean and Civil Engineering, Shanghai Jiaotong University, Shanghai, China; 2 Shanghai Research Centre of Ocean & Shipbuilding Maritime Engineering, China Shipbuilding NDRI Engineering Co. Ltd, Shanghai, China; 3 School of Civil Engineering and Architecture, Guangxi University, Nanning, China; China Construction Fourth Engineering Division Corp. Ltd, CHINA

## Abstract

To address the critical limitations of conventional parallel double-anchor support systems, including coplanar load imbalance, critical spacing risks, deviations from theoretical designs, and maintenance difficulties, this study proposes an innovative multi-anchored sheet pile reinforcement method. Centrifuge model tests incorporating digital image correlation (DIC) techniques were systematically conducted to investigate the anchoring mechanism of the novel system in port basin bulkhead reinforcement, with a particular emphasis on parameter optimization. The results indicate that the excavation depth predominantly governs the mechanical response of quay walls (sensitivity coefficient 2.3), necessitating phased excavation protocols for deformation control. In contrast, tie-rod horizontal spacing demonstrates low sensitivity (0.018), enabling displacement-controlled dynamic optimization to balance safety and cost-effectiveness. Further, strategic anchor rod installation restructures stress transmission paths, effectively suppressing the shear deformation of shallow soil. The synergistic interaction between prestress application and soil–anchor load transfer mechanisms induces potential migration of the slip surface toward reinforced zones, enhancing stability by 34–41% compared to conventional systems. The developed composite prestressed anchor system achieves a 71.9–77.3% reduction in sheet pile bending moments through stiffness enhancement–stress redistribution coupling effects, establishing a new paradigm for high-performance port infrastructure.

## 1. Introduction

With the rapid development of global trade and the surge in demand for deep-water ports, the harbor basin wall engineering not only needs to deal with large water level difference and tidal load, but also needs to solve the problems of low shear strength of soft soil, significant creep effect and difficulty in long-term deformation control [1]. As one of the three primary quay wall structural types [2], sheet pile reinforcement systems have seen extensive implementation in coastal sandy deposits [2–7] due to their structural simplicity, convenience of construction, cost-effectiveness, rapid deployment, and enhanced global stability. The evolution of this technology hinges on innovative structural optimization to improve stability parameters, thereby meeting modern port requirements for mega-scale and deep-water applications. Nevertheless, the design of anchored sheet pile systems lacks robust theoretical foundations, particularly regarding the synergistic mechanisms of multi-anchor configurations, which frequently leads to complications such as anchor load imbalance and deep-seated slip surface propagation. Consequently, developing advanced multi-anchored sheet pile reinforcement techniques for soft soil environments represents both an urgent necessity and a fundamental scientific challenge in the field of geotechnical engineering.

Current engineering practice predominantly involves constructing sheet pile quay walls in favorable geological conditions, while gravity-type quay walls [8–10] and pile-supported wharves [11,12] are typically adopted for soft foundations. The construction of sheet pile structures in soft soil strata faces substantial challenges due to their differential settlement characteristics. International codes (e.g., JTS, OCDI, BS) and existing research primarily focus on single-anchor sheet pile structures [13, 14], with design heories rooted in classical earth pressure distribution assumptions. However, the redistribution of soil stress induced by deep basin excavation and nonlinear soil–structure interactions often leads to anchor overload and insufficient passive resistance in embedded sections for single-anchor systems [15,16]. Field tests by Lu et al. have demonstrated that while maximum horizontal displacements of single-anchor sheet piles in sandy soils remain within design limits, displacement accumulation rates in soft clays increase significantly [17], revealing the limitations of conventional design methods in heterogeneous strata. Furthermore, current codes lack systematic provisions for multi-anchor systems, with only simplified approaches such as the elastic foundation beam method being conceptually referenced [18], resulting in empirical conservative designs in practice. Although multi-anchor configurations theoretically enhance structural stiffness through layered load transfer, their mechanical mechanisms remain inadequately investigated. Fall et al. [19] employed three-dimensional finite element analysis to simulate tunnel construction processes, sheet pile wall installation, prestressed anchor locking, and excavation activities, with the results demonstrating the evolution of displacement and internal force distribution in double-anchored walls. Centrifuge modeling by Khuyen et al. revealed that double-anchored sheet pile walls exhibit superior stability compared to single-anchor systems, reducing lateral displacements, tilt angles, bending moments, and backfill settlements by two-thirds [20]. Despite the enhanced bearing capacity of traditional parallel double-anchor systems over single-anchor configurations, inherent structural deficiencies persist, as follows: (1) Coplanar load transfer-induced stress redistribution—when upper and lower anchors share identical load transfer planes, the reaction forces in lower anchors exceed those in upper anchors by 23−37% [21], generating critical stress gradients. (2) Progressive failure from suboptimal spatial parameters—setting the anchor spacing below a certain critical threshold triggers shear zone propagation at anchor–soil interfaces due to stress superposition effects, elevating system instability risks by 40−60% [19,21,22]. (3) Dual-parameter coupling errors in conventional design—simplified elastic foundation beam models overestimate wall bending moments by 15%−20% while underestimating anchor tensions by 10%−15%, causing structural redundancy misdistribution [23]. (4) Maintenance limitations—deeply embedded parallel anchors hinder post-failure remediation, increasing lifecycle costs by 25%−35%. To overcome these challenges, an innovative multi-anchored sheet pile system integrating upper horizontal tie-rods and lower inclined ground anchors is proposed. The rigid connections of upper tie-rods effectively constrain shallow lateral displacements while bearing active zone loads, enabling operational-phase prestress adjustments and localized repairs to decrease lifecycle costs. Simultaneously, optimized inclination angles of lower anchors redirect load transfer paths, diminishing horizontal shear forces on pile shafts [3]. This dual-mechanism approach achieves synergistic deformation control through load path optimization and stiffness coupling. Unlike conventional parallel systems where coplanar load transfer causes reaction forces in lower anchors to exceed upper ones by 23–37%, the proposed inclined configuration effectively decouples stress concentration zones and mitigates the critical stress gradients typical of traditional designs.

In this study, the new multi-anchor sheet pile centrifuge test was carried out, and the new multi-anchor sheet pile reinforcement system was tracked and captured in real time by using digital image measurement technology. The deformation, failure mode and soil particle displacement cloud diagram of the new multi-anchor sheet pile reinforced harbor bank wall during the loading process were obtained. The influence of anchoring parameters on the anchoring mechanism of the new multi-anchor sheet pile reinforced harbor bank wall was revealed from the macroscopic response and microscopic interaction. It provides a theoretical basis and practical basis for revealing the bearing mechanism of the new multi-anchor sheet pile reinforced harbor bank, evaluating the anchoring effect, and optimizing the design and construction methods.

## 2. Centrifuge model test

### 2.1. Experimental setup and program

**Fig 1:**
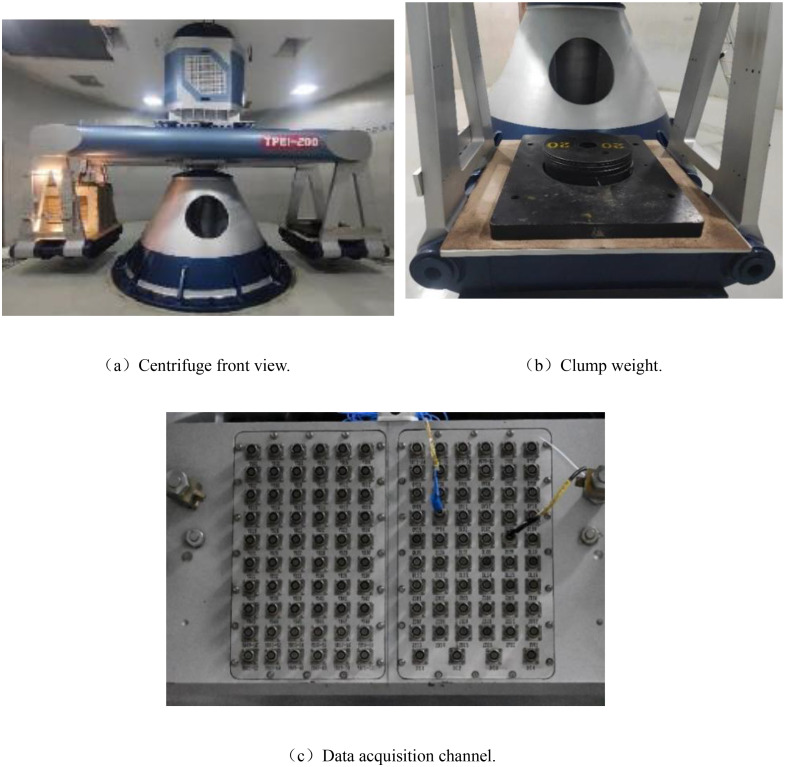
TPEI-200 geotechnical centrifuge.

The centrifuge experiment in this study was conducted using the TPEI-200 geotechnical centrifuge at CCCC Tianjin Port Engineering Institute Co., Ltd, the test conditions are shown in Table 1. The centrifuge has an effective bearing capacity of 200 gt, a maximum centrifugal acceleration of 200 g, and a rotational radius of 4.0 m. The data acquisition system employs a high-precision dynamic-static integrated module with a total of 112 channels, including 80 strain channels, 16 current channels, and 16 vibration channels, as shown in Fig 1. To ensure that the model accurately reflects the prototype’s properties and behavior, the stress state in the centrifugal field must match the stress state of the prototype under gravity while maintaining constant stress [24]. Therefore, the model must satisfy not only geometric similarity, but also similarity in stress and strain. All details regarding geotechnical centrifuge modeling, scaling laws, and principles can be found in Taylor (1994) [25]. The scaling factors relevant to this centrifuge test are listed in Table 2. The dimensions of the centrifuge model box are 1000 mm × 500 mm × 800 mm (length×width×height), with the front face made of transparent plexiglass to allow for observation of soil particle movement around the reinforcement interface during acceleration. A digital image acquisition system is used to perform macro- and micro-analyses of soil particle movement patterns, capturing the progressive deformation behavior of the reinforced soil. The seams of the model box are sealed with welding and adhesive to prevent water loss, which could skew the test results. Based on the principles of similarity, consolidation and settlement analysis, the technical limitations of experimental equipment, and analysis of the pre-test results, a centrifugal acceleration of 60 g was selected to ensure the accuracy, stability, and safety of the test results.

**Table 1:** Summary of test schemes.

Test aim	Exploring the stress and deformation characteristics of components, soil pressure, and overall structural failure modes of various support structures under centrifugal effects.			
Test No.	Test Cond.	Relative density	Position of bolt (cm)	Position of pull rod (cm)
C_1_	Single anchor plate pile	0.65	/	3.3
C_2_	Standard working condition	0.65	8.35	
C_3_	Continued excavation condition	0.65	8.35	
C_4_	Prestressing condition	0.65	8.35	
C_5_	Interlayer condition (5 cm)	0.65	8.35	
C_6_	Thickened soil condition (10 cm)	0.65	8.35	

**Table 2:** Scaling laws for the centrifuge tests.

Experimental parameters		Unit	model: prototype
Soil mass	Density	kg/m^3^	1:1
	Particles	/	1:1
Fundamental	Acceleration	m/s^2^	1:60
	Linear dimension	m	1:60
	Stress	kPa	1:1
	Strain	/	1:1
	Creep	T	1:1
Consolidation	Time	s	1:602
Seepage flow	Coefficient of seepage	m/s	1:60
	Time	s	1:602
Dynamic load	Vibration velocity	m/s	1:1
	Vibration frequency	s^-1^	1:60
	Vibration time	s	60
Structural member	Axial force	N	602
	Bending moment	N·m	603
	Axial stiffness (EA)	N	602
	Flexural stiffness (EI)	N·m^2^	604

### 2.2. Test materials and model preparation

#### 2.2.1. Test materials.

**Fig 2:**
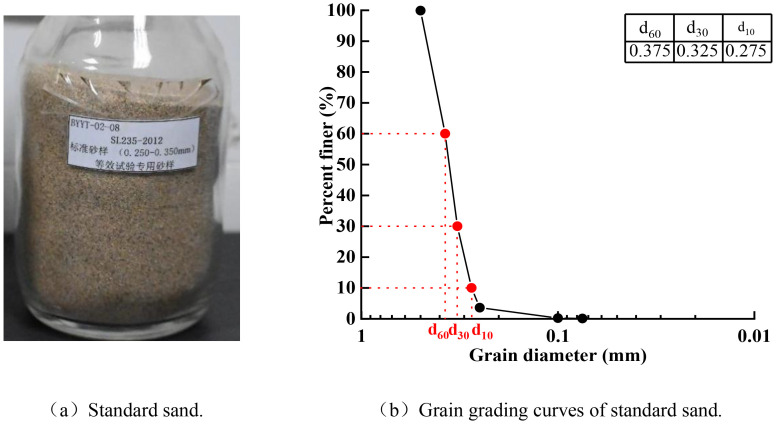
Standard sand and grading curve.


The test soil consisted of standard Fujian sand and kaolin. The standard sand, a commonly used material in model testing apparatus for element tests, underwent conventional physical and mechanical tests in accordance with the Chinese Standard for Geotechnical Testing Methods (GB/T 5013-2019) [26]. The particle size distribution curve of the sand is shown in Fig 2(b), with its characteristic particle sizes and parameters summarized in Table 3. For prototype structures, the CAZ24-700 steel sheet piles measured by ISO 148-1 [27], GB/T 228.1–2021 [28], ASTM E111-17 [29]. Its bending stiffness (EI) is 6.07 × 10 Ω kN· m 2/ m. The prototype anchor sheet pile adopts C30 concrete, and the elastic modulus is 3.0 × 10⁴ MPa, which meets the design requirements of tensile stiffness (EA) and bending stiffness. In the scaled model (1:60), SUS304 stainless steel tubes with dimensions of 7 mm width and 1 mm thickness were used to simulate anchor sheet piles, arranged at 12.5 cm intervals. The model’s flexural rigidity satisfied computational requirements through similarity principle verification. The prototype pile cap, constructed with C30 concrete measuring 20 m×1.0 m×1.6 m (length×width×height) and an elastic modulus of 3.0 × 10¹⁰ Pa, was replicated using rectangular aluminum plates (density, 2.69 g/cm³; elastic modulus, 6.8 × 10¹⁰ Pa; shear modulus, 2.5 × 10¹⁰ Pa; Poisson’s ratio, 0.32). Based on the equivalence of flexural rigidity, the model cap dimensions were designed as 498 mm × 40 mm × 2 mm (L×W×H). Original ground anchors consisting of four φ19.5 mm steel strands (24 m length, 500 mm borehole diameter) and grouted with C30 cement mortar (elastic modulus Ep = 2.0 × 10¹⁰ Pa) were modeled using phosphor bronze strips (7 mm width × 400 mm length × 3 mm thickness) at a 1:60 scale. This substitution enabled strain gauge attachment for moment measurement. Through the rigorous application of EI equivalence principles, combined with Equations (1) and (2), critical model dimensions were determined as 5.5 mm for sheet piles, 6 mm for anchor piles, 1 mm for tie-rods, and 3 mm for anchors, ensuring mechanical similarity with prototype components.

**Table 3:** Basic properties of soil.

Soil type	Property	Value
Standard sand soil	Ρdmax (g/cm3)	1.78
	Ρdmin(g/cm3)	1.47
	Specific gravity	2.65
	Φ(°)	39.6
	c(kPa)	9.3
Soft soil	γ (kN/m3)	18.4
	K (cm/s)	1.21 × 10−6
	M(MPa)	3.01
	e	1.14
	Φ(°)	39.6
	c(kPa)	19.2

φ, internal friction angle; γ, unit weight; k, permeability coefficient; M, modulus of compression; e, void ratio.

$$E_{m}I_{m}=n^{-4}E_{p}I_{p}$$ (1)

Ip=bh3/bh312\nulldelimiterspace12 (2)

#### 2.2.1. Test materials Soil sample preparation.

**Fig 3:**
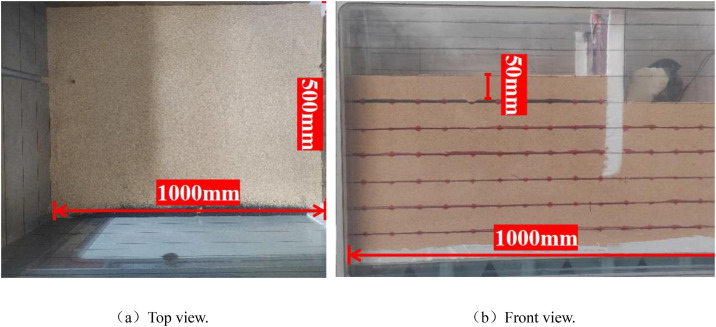
Photograph of model box.

To minimize systematic errors induced by excessive acceleration and inconsistent linear velocities, the geometric scale ratio was determined as N = 60 after the comprehensive consideration of model dimensions, material properties, and sensor configuration [30,31]. The model container dimensions are illustrated in Fig 3. Prior to soil placement, the inner walls of the container were coated with Vaseline to mitigate boundary effects caused by soil–container wall friction.

The soil was placed in 12 layers with 50 mm thickness per layer. A 10 mm-wide × 3 mm-thick colored sand strip was embedded along the transparent glass face (Fig 3(b)) for displacement monitoring. The sand was deposited using the sand raining method and compacted through the layered compaction technique [32], with each layer leveled using a straightedge before subsequent placement. This procedure ensured uniform material distribution and maintained relative density at approximately 0.65. For soft soil layer construction, 200 mm-thick layers were implemented. Prior to placement, five moisture content measurements were conducted, with deviations controlled within 2% of the target value. After reaching the specified elevation, the surface was leveled and subjected to vertical surcharge loading to accelerate consolidation. Spatial homogeneity was verified using cone penetrometer testing, preventing differential settlement that might compromise the experimental results during subsequent sand layer placement.

### 2.3. Measuring instrument

**Fig 4:**
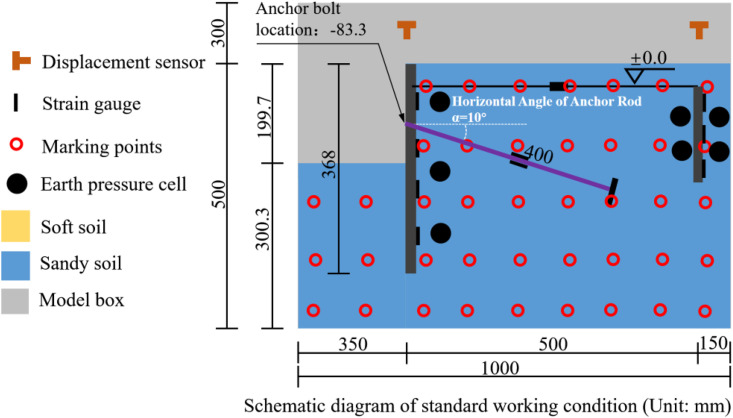
The overall distribution position of each overall sensor.

This study evaluated the mechanical response of a novel multi-anchor plate pile support system in harbor basin wall reinforcement through centrifuge model tests, with a focus on monitoring the horizontal displacement field of steel sheet piles, the stress field at soil–structure interfaces, the force transmission mechanism of tie-rods, and the evolution of soil displacement. The monitoring system integrates GK4000 strain gauge, ZXPSO-01 earth pressure sensor, DME5000-221 laser displacement sensors, and digital image correlation (DIC) technology to establish a multi-physical field-coupled measurement system. The primary sensor locations are shown in Fig 4.

Six layers of strain monitoring arrays (for steel sheet piles) and three layers (for anchor plates) were established in key mechanical response zones, with interlayer spacings of 50 mm. A full-bridge Wheatstone bridge configuration (using 120 Ω foil strain gauges) was employed for dual-sided symmetric installation, and dynamic strain data were collected using an optical fiber Bragg grating demodulator (resolution ±1 με). After sensor installation, high-modulus epoxy resin (elastic modulus 3.5 GPa) was applied for encapsulation and protection, ensuring a continuous strain transfer interface with the 6061-T6 aluminum alloy substrate. Miniature earth pressure sensors (MEPS, range 0-500 kPa, non-linearity ≤ 0.5%FS) were installed along the soil–structure interface normal direction at intervals of 50 mm. A voltage–earth pressure conversion matrix was established through pre-test calibration [33], and the theoretical earth pressure curve was fitted using the least squares method to eliminate measurement deviations caused by interface contact stiffness differences. A three-dimensional laser displacement sensing system (DS, sampling rate 1 kHz, accuracy ±0.05%FS) was used to synchronously monitor the spatial displacement fields of steel sheet piles and anchor plates. A non-contact displacement monitoring system was established based on particle image velocimetry (PIV); artificial tracer particle layers (particle size 0.2-0.5 mm) were pre-installed within the model cross-section, and a high-speed CMOS camera (2000 fps, resolution 4096 × 2160) was used to continuously capture soil deformation sequence images. Sub-pixel cross-correlation algorithms (window size 32 × 32 pixels) were employed to calculate displacement gradient tensors and, combined with digital image correlation (DIC) technology, displacement data were sampled at a rate of 1 sample per second [34,35], enabling real-time measurement of the entire soil particle displacement process and the reconstruction of soil displacement cloud maps.

### 2.4. Test procedures and conditions

**Fig 5:**
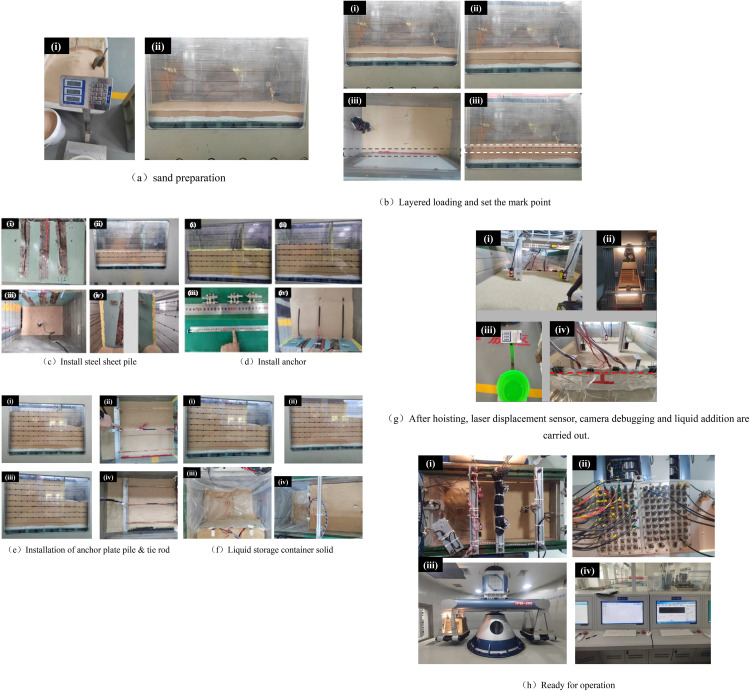
New multi-anchor sheet pile centrifugal model making process.

The main procedures of the centrifuge test are summarized as follows (Fig 5):

(1) Soil sample preparation—Dry standard Fujian sand was used. Based on the relative density, the mass of sand for each layer was determined, with 36.2 kg required for every 50 mm of height. For 10 layers, a total of 362 kg was initially required. However, 50.4 kg (12.6 kg × 4) was deducted for the excavation portion, resulting in a final total mass of standard sand of 311.6 kg;
(2) Marking lines on the model box—According to the standard conditions, the height of each sand layer (50 mm) and the installation positions of steel sheet piles, anchor plate piles, tie-rods, and anchor bars were marked;
(3) Sensor assembly on components—Strain gauges and earth pressure sensors were attached to the corresponding positions on steel sheet piles, tie-rods, anchor bars, and anchor plate piles. The subsequent calibration of these components was performed;
(4) Sand layer preparation—Sand layers were compacted to designed elevations through sequential placement. Each layer was leveled, marked with reference points, and covered with colored sand;
(5) Component installation—Upon reaching designated elevations during sand placement, steel sheet piles, anchor rods, anchor slab piles, and tie rods were installed sequentially. Particular attention was paid to gaps between steel sheet piles and the model box. These gaps were sealed using modeling clay or geotextile membranes to prevent sand leakage during centrifuge operation;
(6) Drainage system installation—Liquid storage containers were connected to solenoid valves via quick-connect couplings. Containers were organized and aligned, with geotextile fabric attached to the excavation side of steel sheet piles to prevent puncture damage from sharp edges;
(7) Sensor configuration—Laser displacement sensors were positioned strategically. All sensors were connected to the centrifuge’s data acquisition channels;
(8) Liquid filling—Based on the approximate conversion of excavated soil mass, 35 kg of liquid was added to the drainage system to simulate excavation-induced mass removal;
(9) System calibration—Strain gauges and laser displacement sensors underwent final calibration checks prior to testing;
(10) Test execution—Each test group was incrementally loaded at 10 g intervals. After reaching 60 g acceleration, the system was maintained at 60 g for 3 minutes to achieve stability. The simulated excavation was then initiated through the controlled operation of solenoid valves.

## 3. Test result analysis

### 3.1. Displacement variation characteristics

**Fig 6:**
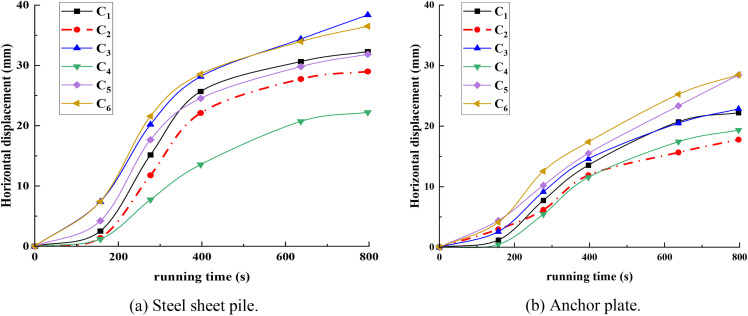
Displacement vector evolution diagram of stone columns.

The nonlinear deformation characteristics of the soil–structure interaction system were revealed through displacement time–history curves of steel sheet piles and anchor slab piles under six working conditions (C_1_–C_6_), as shown in Fig 6. Notably, Case C_6_ exhibited the maximum average horizontal displacement of 32.5 mm for both steel sheet piles and anchor slab piles, significantly exceeding other cases. In contrast, the minimum average horizontal displacement (20.8 mm) was observed in another case, representing a 36% reduction compared to the maximum value, indicating enhanced system stiffness. Monitoring data demonstrate that the horizontal displacement evolution of the supporting structure follows a typical three-stage pattern, with distinct mechanical mechanisms, as follows: Stage I—Deformation Coordination Phase (0 < t ≤ 200). The displacement growth rate decreases, accompanied by increased dispersion among case curves. This phenomenon is attributed to the progressive activation of support system stiffness. The composite constraint effect—The sequential mobilization of support components (e.g., anchor rods, tie rods) generates synergistic constraints. Soil energy dissipation—Concurrently, the soil enters an alternating shear dilation and compaction phase, dissipating energy to mitigate structural deformation. Stage II—Plastic Development Phase (200 < t ≤ 600). Continued excavation triggers rapid stress redistribution. Stress release—A sharp reduction in confining stress around the supporting structure. Lateral thrust intensification—Significant increases in soil lateral thrust and structural loading. Deformation coupling—Pronounced shear and volumetric deformations during adaptation to new stress states amplify lateral pressures, driving accelerated horizontal displacement. Stage III—System Stabilization Phase (t > 600). Horizontal displacement stabilizes with minimal increments due to stress equilibrium (the completion of soil stress redistribution establishes a new balance between the structure and surrounding soil). Stiffness mobilization—Uniform internal stress distribution and the full activation of structural stiffness substantially reduce further deformation potential. Plastic zone stabilization—The fixed extent of soil plastic zones and completion of shear/volumetric deformations result in negligible increases in lateral soil–structure interaction forces.

### 3.2. Soil pressure on both sides of the steel sheet pile

**Fig 7:**
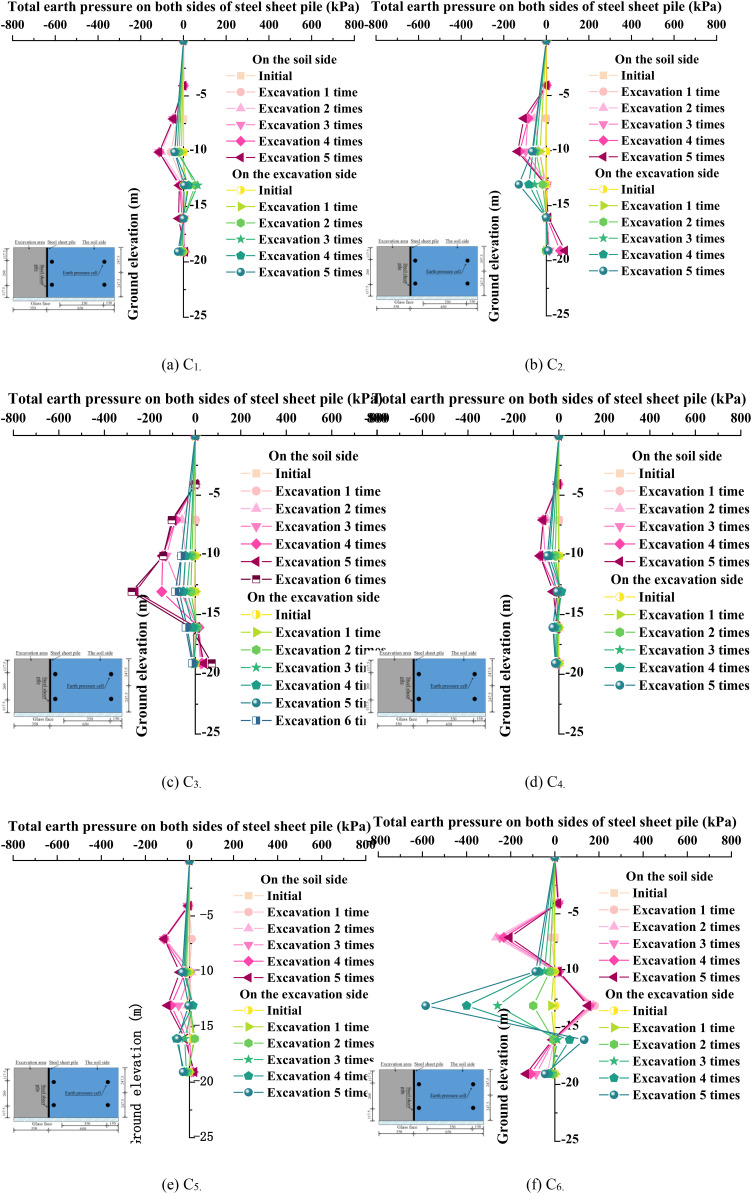
Variation characteristics of horizontal earth pressure on both sides of steel sheet piles.

The horizontal earth pressures were measured using pressure sensors installed on both sides of the steel sheet pile. Fig 7 illustrates the variation patterns of horizontal earth pressures on both sides of the steel sheet pile during excavation under different working conditions. The results demonstrate that the earth pressures on both sides exhibited nonlinear variations during excavation, with values increasing progressively with excavation stages. The transition of earth pressures from negative to positive values corresponds to the gradual reduction in excavation depth, indicating the coexistence of both passive and active earth pressures on opposite sides of the sheet pile, with one side compressing the soil mass while the other side separates from the retained soil. Comparative analysis between Fig 7(a) and 7(b) reveals that anchor installation significantly amplifies the earth pressure variations in the retained soil zone, where the excavation-side earth pressure predominantly manifests as passive earth pressure. This phenomenon can be interpreted as the combined effect of anchors and anchor cables inducing passive earth pressure above the excavation base level, while the sub-excavation zone experiences compressive stresses toward the excavation side, further confirming passive pressure characteristics. This demonstrates that the anchor implementation effectively modifies the earth pressure distribution. The comparison between standard and prestressed working conditions in Fig 7(b) and 7(d) shows that after five excavation stages, the peak earth pressures at -10 m elevation were -134.42 kPa and -82.45 kPa for the retained side, and -129.03 kPa and -26.39 kPa for the excavation side, under respective conditions. The application of prestress reduced peak pressures by 38.7% and 79.5% on the retained and excavation sides, respectively. This phenomenon is primarily attributable to the early application of prestress, which effectively offsets part of the stress concentration caused by soil displacement and excavation. Therefore, applying prestress can significantly enhance the safety and cost-effectiveness of the support system, providing an important reference for the design optimization of foundation pit projects under complex conditions. The comparative analysis of Figs 6(b), 6(e), and 6(f) reveals distinct pressure distribution patterns—a “V”-shaped configuration under standard conditions evolves into an “M”-shaped pattern with a 3 m soft soil interlayer, and finally transforms into a composite “V”-shaped pattern with a 6 m soft soil interlayer. After five excavation stages, the peak earth pressures at -10 m elevation were measured as -134.43 kPa, -43.91 kPa, and 17.07 kPa for these three conditions, respectively. Comparing Figs 6(b), 6(e), and 6(f), it can be observed that the pattern of soil pressure changes from a “V”-shaped pattern under standard conditions to an “M”-shaped pattern with a 50 mm soft soil interlayer, and finally to a “V”-shaped spliced pattern with a 100 mm soft soil interlayer. After five excavations under the three conditions, the peak soil pressures at an elevation of −10 m were −134.43 kPa, −43.91 kPa, and 17.07 kPa, respectively. Due to the increased excavation depth, the sheet piles experienced horizontal displacement toward the excavation side, causing anticlockwise rotation relative to the embedded ends of the sheet piles. This resulted in the passive squeezing of the soil below the excavation base elevation. After five excavations, the peak soil pressures at an elevation of -12 m were -129.03 kPa, -3.59 kPa, and -585.17 kPa, respectively. As the thickness of the soft soil interlayer increased, the horizontal displacement and anticlockwise rotation of the sheet piles significantly intensified, leading to an abnormal increase in soil pressure in the region below the embedded ends.

### 3.3. Soil pressure on both sides of the anchor sheet pile

**Fig 8:**
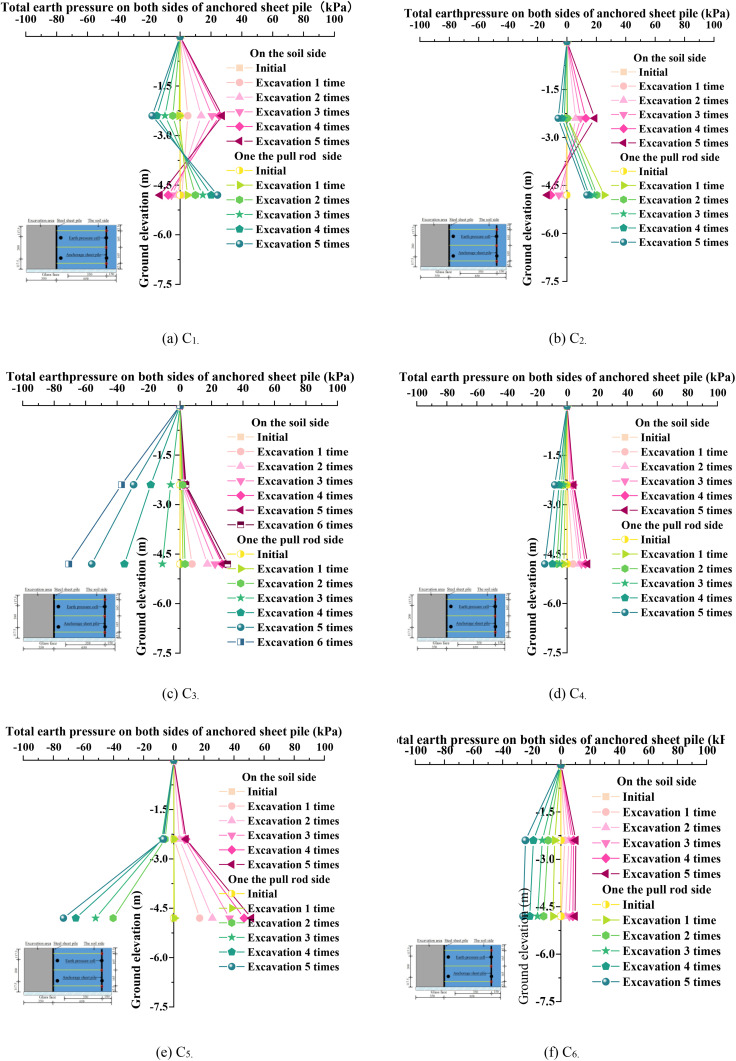
Variation characteristics of horizontal earth pressure on both sides of anchor sheet pile.

Fig 8 presents the soil pressure variation curves on both sides of the anchor plate piles during excavation under continued deepening conditions. As depicted in Fig 8, the soil pressures on both sides of the anchor plate piles exhibited nonlinear changes and increased with the number of excavations. The transition points where the soil pressure on the rod side shifts from negative to positive (or vice versa on the soil side) correspond to progressively decreasing excavation depths. This indicates that, during each excavation phase, both passive and active soil pressures are present on either side of the anchor plate piles. As the soil pressure cells are arranged along the length of the anchor plate piles and are symmetrically positioned on both the rod and soil sides, the soil pressure distribution in Fig 8(b) also demonstrates a symmetric pattern. The maximum positive and negative soil pressures were 30 kPa and −70 kPa, respectively, which differ by 8 kPa and −24 kPa from those observed in Fig 8(a). Comparing Fig 8(a) and 8(b), it is evident that as excavation progresses, the horizontal soil pressures on both sides increase nonlinearly. This phenomenon is primarily due to the unloading of the soil mass, leading to a redistribution of the original stress field. The vertical stress below the excavation face decreases, while the horizontal stress is released due to the lateral expansion of the soil mass. However, the presence of the support structure limits the free deformation of the soil, preventing the horizontal stress from fully releasing to the active limit state. Consequently, the actual soil pressure lies between the static and active soil pressures and, as the excavation deepens, the cumulative effect of lateral stresses that the support structure must bear becomes increasingly significant. After the addition of anchor rods, as excavation progresses, the total soil pressure on the soil side gradually decreases, while the negative soil pressure on the rod side increases. This indicates that excavation weakens the soil’s ability to support the piles while simultaneously increasing the tensile demand on the anchor rods. Comparing Fig 8(b) and 8(c), it is evident that with the continued deepening of excavation, the maximum horizontal soil pressure on the excavation side and the horizontal soil pressure on the anchor rod side increased from 18.9 kPa and 25.4 kPa to 30.2 kPa and 70.7kPa, representing increases of 59.8% and 178.4%, respectively. The significant rise in horizontal soil pressure on the anchor rod side can be attributed to substantial deformations caused by deep excavation, which induce further soil relaxation and sharply increase the tensile demand on the anchor rods. When prestress was applied to the anchor rods under the C_2_ condition, as shown in Fig 8(b) and 8(d), the horizontal soil pressures on both the excavation and anchor rod sides became more symmetric, with the maximum horizontal soil pressures decreasing to 14.9 kPa and 13.7 kPa, corresponding to reductions of 21.2% and 46.1%, respectively. This improvement is primarily because prestressed anchor rods apply reverse tensile forces that suppress the unloading effect in the active soil region, causing the active soil pressure to approach the static soil pressure state. Additionally, the pretension densification effect enhances the stiffness and strength of the passive soil region, optimizing passive resistance. Moreover, the synergistic interaction between the anchor rods and the support structure increases the overall system rigidity, restricts soil deformation, and redistributes the stress field, promoting a more symmetric distribution of soil pressures on both sides. Consequently, through the coupled effects of mechanical balance, deformation coordination, and load redistribution, soil pressures are efficiently regulated, thereby enhancing engineering safety. Comparing Fig 8(e) and 8(f), it can be observed that under the C_5_ and C_6_ conditions, the horizontal soil pressures on the excavation and anchor rod sides increased from 25.9kPa and 9.7kPa to 73.3kPa and 51.7kPa, representing increases of 183.0% and 432.9%, respectively. This substantial rise is primarily due to the increased thickness of the soft soil layers, which leads to load superposition effects, resulting in the higher self-weight stress of the soil mass and the extension of the sliding surface. Consequently, more soil undergoes sliding, and the increase in horizontal soil pressure exhibits a nonlinear trend. Additionally, the interaction between the support system and the soft soil deteriorates; after the soft soil layers are thickened, portions of the anchor rod anchorage sections are found to reside within weaker soil layers, significantly reducing their uplift resistance and leading to a decline in the anchorage performance of the anchor rods.

### 3.4. Steel sheet pile bending moment

**Fig 9:**
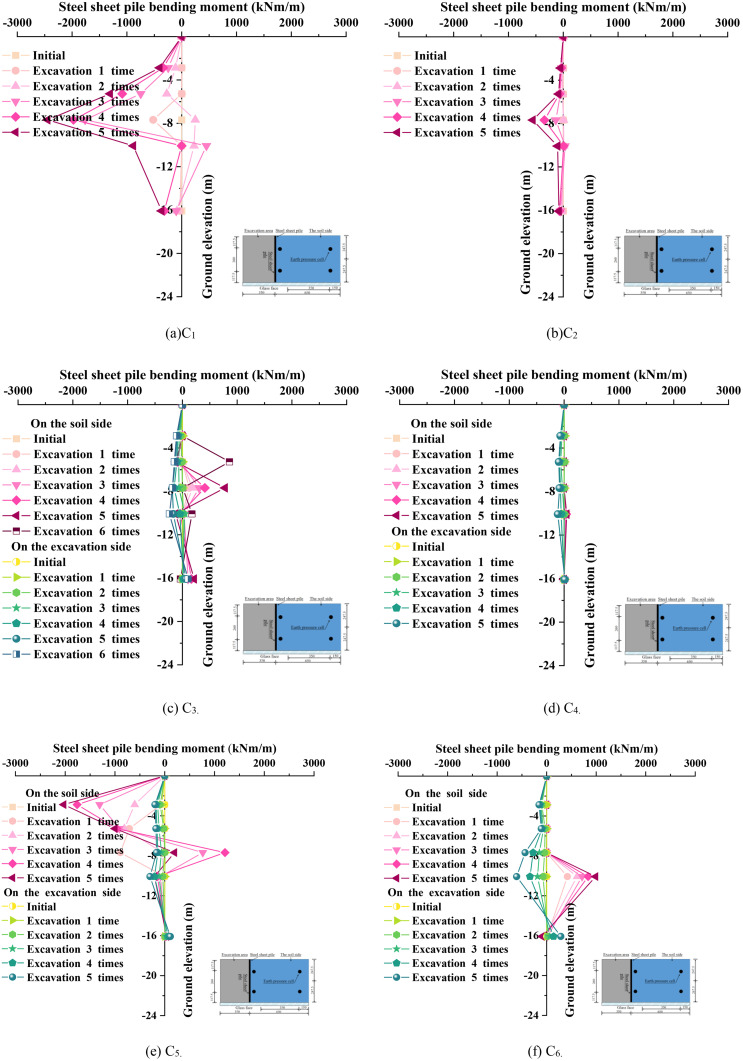
Variation law of the bending moment of a steel sheet pile.

Fig 9 illustrates the distribution curves of bending moments in steel sheet piles under different operating conditions. Under conditions C_1_ and C_2_, the bending moments on the excavation side and the soil side are symmetrically arranged based on strain gauges, without distinguishing their directional differences. A comparative analysis of Fig 9(a) and 9(b) reveals that, with the implementation of anchor rod reinforcement (condition C_2_), the maximum bending moment in the steel sheet piles significantly decreased from −2447 kNm/m in condition C_1_ to −555 kNm/m in condition C_2_, representing a reduction of 77.3%. This phenomenon indicates that the anchor rod system enhances the overall stiffness of the structural system through anchorage effects, effectively suppressing the structural deformation induced by soil lateral pressures and thereby achieving a substantial reduction in bending moments. Building upon condition C_2_, when the excavation depth increased by 50 mm to form condition C3 (Fig 9(c)), the maximum bending moment rose to 778.8 kNm/m, marking an increase of 40.3% compared to condition C_2_. This highlights the significant impact of soil unloading effects on the structural stress distribution. Conversely, when prestress was applied to the anchor rods under condition C_2_ to form condition C_4_ (Fig 9(d)), the prestress system effectively constrained the lateral displacement in the region above the structural neutral axis. Under these circumstances, the maximum negative bending moment on the excavation side decreased to −155.97 kNm/m, and the positive bending moment on the soil side decreased to 46.34 kNm/m, both reductions of 71.9% compared to condition C_2_. This confirms that prestressing technology, through the establishment of an active stress field, significantly improves the stress distribution within the structural system.

This study demonstrates that the synergistic effects of anchor rods and prestressing can form a composite support system. Through the coupling of stiffness enhancement mechanisms (anchor rods) and stress regulation mechanisms (prestressing), it is possible to effectively reduce the bending moment responses of steel sheet pile structures by 71.9% to 77.3%. This performance constitutes a significant improvement over conventional elastic foundation beam models, which typically overestimate bending moments by 15%–20% but fail to achieve such substantial structural unloading. This has significant engineering application value for the safety control of deep foundation pit projects.

### 3.5. Axial force of pull rod

**Fig 10:**
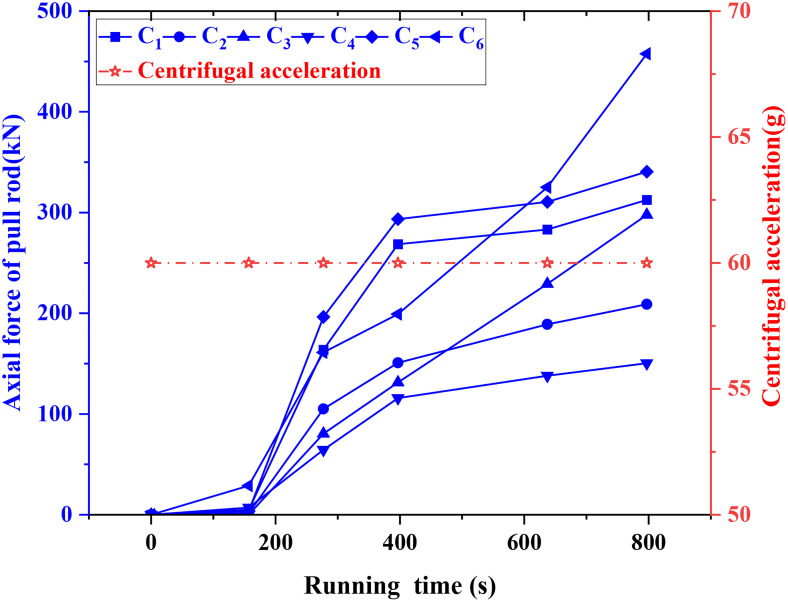
Change rule of rod axial force.

Fig 10 shows the axial mechanical properties of the lower pull rod under six working conditions (C_1_-C_6_), and reveals the law of force under different engineering conditions: the single anchor sheet pile (C_1_) has no anchor rod, and the stiffness of the supporting system is low. The passive earth pressure cannot be effectively transmitted, and the pull rod needs to bear all the horizontal loads. The pull rod shows a high axial force (313 kN), which is suitable for light load scenarios. The standard operating condition (C_2_) is used as the reference value (209 kN) to reflect the conventional design; the continuous unloading of soil in continuous excavation (C_3_) leads to the accumulation of displacement, which leads to a reduction in effective stress in the anchorage zone and the degradation of the frictional resistance of the contact surface. The change of stress path forced the pull rod to bear the new soil slip thrust, which increased the axial force to 298 kN. Dynamic monitoring during construction was needed to control the risk of additional load. The maximum load of the pull rod was 150 kN, induced by preloading (C_4_), and the subsequent load growth rate was slow, indicating that the preloading produced a resistance reserve through the initial compression of the soil, which partially offset the active earth pressure increment caused by subsequent excavation. The interfacial shear band is formed in the thin soil layer (C_5_) due to the sudden change of modulus, and the dilatancy effect of the soil aggravates the local stress redistribution. The interface slip leads to the failure of the anchorage section constraint, and the load is transferred to the tie-rod (341 kN), which needs to be prevented from local instability. Due to the superposition of the soil pressure of 10 cm to the peak value of 458 kN, high-strength materials or composite anchorage schemes are required for the thickened soil (C_6_). The stable 60 g centrifugal environment in the test ensures the reliability of the data, and the force of the pull rod shows a nonlinear law of slow increase in the early stage (0–200 seconds) and steep rise in the later stage (after 400 seconds). It is suggested that prestress should be used to optimize the initial stress, strengthen the structural design of thick soil/interlayer conditions, and expand the research on soil parameters, long-term load, and field verification, so as to provide theoretical support for the safety design and risk control of practical engineering.

## 4. Sensitivity analysis of reinforcement parameters

**Fig 11:**
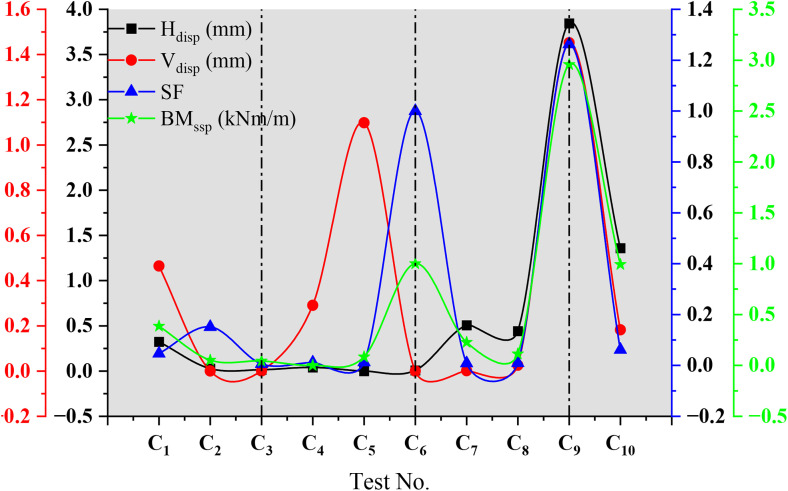
Summary chart of test results for each group. Note: Hdisp= horizontal displacement; Vdisp= vertical displacement; SF= safety factor; BMssp= bending moment of steel sheet pile.


The test results of the previous content are similar, and limited to space, only the test results of C_1_-C_6_ conditions are given. Through the geotechnical centrifuge model test, the effects of important factors such as horizontal spacing of tie rods, stiffness of steel sheet piles and prestress on the stability of the bank wall of the harbor basin were studied. In order to comprehensively analyze the sensitivity analysis of the influence parameters, all the test results are shown in Fig 11. For the model test of the new multi-anchor sheet pile used to reinforce the bank wall of the harbor basin, the system characteristics *P* (e.g., horizontal displacement, steel sheet pile bending moment, etc.) can be expressed as a function that determines the system parameters (e.g., the horizontal spacing of tie-rods, the stiffness of steel sheet piles, and prestress) [36]. The functional expression between system characteristics and factors is as follows:

$$P=f(a_{1},a_{2},\cdots \;,a_{k},\cdots \;,a_{n})$$ (3)

To partially mitigate scale effects and achieve a more effective qualitative representation, the experimental results were nondimensionalized to facilitate a horizontal comparison of sensitivity levels among various factors. Accordingly, the sensitivity function $S_{k}(\alpha_{k})$ of parameter $\alpha_{k}$ is defined as the ratio of the relative error δp=|ΔP|/|ΔP|P\nulldelimiterspaceP of the system characteristic (*P*) to the relative error δak=|Δak|/|Δak|ak\nulldelimiterspaceak of parameter $a_{k}$,


Sk(ak)=(|ΔP|P)/(|ΔP|P)(Δakak)\nulldelimiterspace(Δakak)=|ΔPΔak|akPk=1,2,⋯,n (4)

When |Δak|/|Δak|ak\nulldelimiterspaceak is small enough, $S_{k}(\alpha_{k})$ can be approximately expressed as


$$S_{k}(a_{k})=\left|\frac{d\varphi_{k}\left(a_{k}\right)}{da_{k}}\right|\;\frac{a_{k}}{P}\;\;\;\;\;\;\;\;\;\;\;k=1,\;\;2,\;\cdots ,\;\;\;n$$ (5)

The sensitivity function curve $S_{k}-\alpha_{k}$ of $a_{k}$ can be plotted using Equation (5), where $\alpha_{k}=\alpha_{k}^{*}$. The sensitivity factor $S_{k}^{*}$ of $a_{k}$ is then obtained as


$$S_{k}^{\;*}=S_{k}(a_{k}^{\;*})=\left|{\left[\frac{d\varphi_{k(a_{k})}}{da_{k}}\right]}_{a_{k}=a_{k}^{\;*}}\right|\;\frac{a_{k}^{\;*}}{P}\;\;\;\;\;\;\;\;\;\;k=1,\;\;2,\;\cdots ,\;\;\;n$$ (6)

The $S_{k}^{*}$ value exhibits a positive correlation with the sensitivity of *P* to $a_{k}$, indicating that a higher $S_{k}$ value signifies a greater influence of the factor on the system characteristics. Sensitivity function curves of $S_{k}$ versus $a_{k}$ for various factors were generated by extracting data from Fig 11, and the parameter sensitivity factors $S_{k}^{*}$ are presented in Table 4. Furthermore, to elucidate the contribution of each factor to the overall structural stability and to provide clear guidance for design optimization, a comprehensive weighting evaluation method was employed. This approach incorporates the importance of different indicators into the evaluation system, thereby facilitating a more scientific approach to guiding engineering strategies.

**Table 4:** Parametric sensitivity analysis of the load-bearing performance of novel multi-anchored sheet piles.

Test No.	Parameter	Sensitivity of Load-Bearing Performance				
Hdisp	Vdisp	SF	BMssp	W_i_
C_1_	SSPS	3.23 × 10^−1^	4.65 × 10^−1^	4.66 × 10^−2^	3.86 × 10^−1^	2.5 × 10^−1^
C_2_	SSPPR	2.69 × 10^−2^	5.18 × 10^−4^	1.51 × 10^−1^	5.11 × 10^−2^	8.1 × 10^−2^
C_3_	HSTR	1.34 × 10^−2^	5.07 × 10^−4^	5.90 × 10^−3^	4.27 × 10^−2^	1.8 × 10^−2^
C_4_	TRSR	3.97 × 10^−2^	2.91 × 10^−1^	1.17 × 10^−2^	2.47 × 10^−7^	4.2 × 10^−2^
C_5_	APRR	4.25 × 10^−5^	1.09 × 10^0^	1.23 × 10^−2^	8.31 × 10^−2^	4.0 × 10^−2^
C_6_	APRSR	7.21 × 10^−3^	1.51 × 10^−4^	1.00 × 10^−0^	1.00 × 10^−0^	7.0 × 10^−1^
C_7_	HASP	5.05 × 10^−1^	1.83 × 10^−3^	8.19 × 10^−3^	2.27 × 10^−1^	5.0 × 10^−1^
C_8_	APS	4.41 × 10^−1^	2.60 × 10^−2^	9.41 × 10^−3^	1.13 × 10^−1^	1.3 × 10^−1^
C_9_	DDD	3.84 × 10^−0^	1.45 × 10^−0^	1.26 × 10^−0^	2.95 × 10^0^	2.3 × 10^−0^
C_10_	TSSL	1.35 × 10^−0^	1.82 × 10^−1^	6.19 × 10^−2^	9.93 × 10^−1^	6.1 × 10^−1^

Note: SSPS = Steel sheet pile stiffness. SSPPR = Penetration ratio of steel sheet pile. HSTR = Horizontal spacing of tie-rods. TRSR = Stiffness ratio of tie-rods. APRR = Insertion ratio of anchor plate. APRSR = Stiffness ratio of anchorage plate. HASP = Horizontal gap of anchor pile. APS = The prestress of the anchor. DDD = Excavation depth of harbor basin. TSSL = Soft soil interlayer thickness.


$$W_{i}=\overset{n}{\underset{j=1}{\Sigma }}\left(\left|S_{ij}\right|\cdot P_{j}\right)$$ (7)

Among them, S_ij_ is the sensitivity coefficient of the i th factor to the *j* th index, and P_j_ is the weight of the *j* th index. According to the actual needs of the project, the weights of BMssp, Hdisp, SF, and Vdisp are 0.3, 0.2, 0.4, and 0.1, respectively.

The results indicate that, for Hdisp, Vdisp, SF, and BMssp, the ranking of sensitivity factors from the largest to smallest among the top four factors is as follows: S* DDD > S* TSSL > S* HASP > S* APS; S* DDD > S* APRR > S* SSPS > S* TSSL; S* DDD > S* APRR > S* SSPS > S* TSSL; S* DDD > S* APRSR > S* TSSL > S* SSPS. Among various influencing factors, including harbor pool excavation depth, soft soil interlayer thickness, anchorage plate stiffness ratio, and steel sheet pile stiffness, the harbor pool excavation depth has the greatest impact on the characteristics of the harbor pool wall. During the reinforcement of the harbor pool, it is crucial to control the excavation depth, either by minimizing it or employing staged excavation to mitigate the negative effects of excessive depth on structural stability. On the other hand, the sensitivity coefficient of tie-rod horizontal spacing is the smallest, indicating its limited influence on overall stability, allowing for the appropriate optimization of its layout. When harbor pool excavation cannot be avoided, enhancing the anchorage plate stiffness ratio can effectively improve the overall stability of the harbor pool wall. Additionally, the dynamic adjustment of different influencing factors can be undertaken to adapt to various engineering requirements. For instance, in projects located in soft soil areas where foundation deformation control becomes a critical design consideration, particular attention should be paid to controlling structural horizontal displacement. Through sensitivity analysis, when the harbor pool excavation depth cannot be altered, the key factor with the greatest impact on horizontal displacement is identified as the tie-rod horizontal spacing. The appropriate optimization of tie rod horizontal spacing design can be implemented, such as reducing spacing to enhance anchoring effectiveness, thereby improving the harbor pool wall’s resistance to horizontal displacement and ensuring the overall stability and safety of the structure. This method of dynamically adjusting design parameters effectively enhances the adaptability and safety of the structure, making it particularly important for engineering design under complex geological conditions.

## 5. Analysis of failure mode

**Fig 12:**
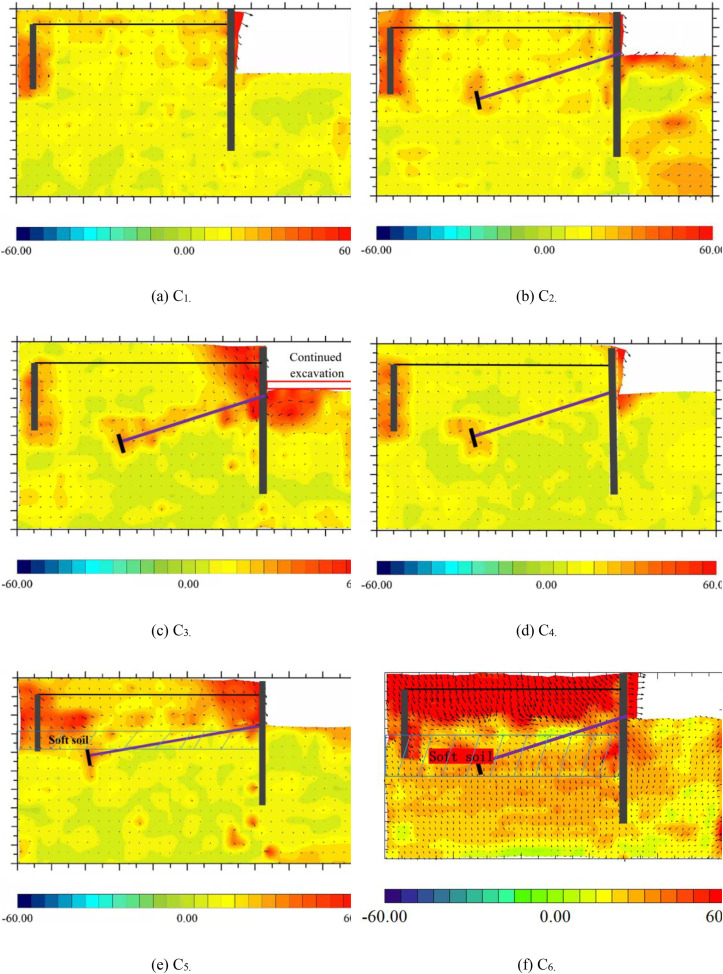
Failure mode of supporting structure.

Through systematic comparisons of failure mechanisms under different working conditions, this study reveals the critical controlling effects of bolt confinement, prestress loading, and geological heterogeneity on the evolution of failure modes. As shown in Fig 12(a) and 12(b), the addition of bolts resulted in a significant reduction in total incremental displacement and caused failure surfaces to migrate toward the anchored zones, confirming that bolts enhance the lateral confinement of the soil, thereby optimizing stress transfer pathways and effectively suppressing shallow shear deformation. The further analysis of deep excavation conditions (Fig 12(c)) reveals that, although the continued excavation of 50 mm after bolt installation increased total displacement to 20 times that of the original condition, the failure surface depth significantly shifted downward and was accompanied by the expansion of shear zones in deeper soil layers. This mechanism is attributed to the passive confinement of deeper soil layers by bolts, which causes potential slip surfaces to extend toward deeper regions, indicating the spatial control capability of bolts over failure modes. While conventional parallel systems risk triggering deep shear zone propagation when anchor spacing falls below critical thresholds—elevating instability risks by 40%–60%—the novel system forces the potential slip surface to migrate toward the reinforced zones. This mechanism enhances global stability by 34%–41%, effectively converting deep-seated failure risks into controllable localized deformation. Under the prestress condition (Fig 12(d)), both total incremental displacement and soil displacement around the tie-rods decreased synchronously, due to the load-sharing effect of prestressed bolts through their axial force transfer mechanism, which induced stress redistribution in the soil and enhanced overall structural stability. Additionally, the comparison of working conditions with 50 mm and 100 mm soft soil interlayers (Fig 12(e) and 12(f)) shows that total displacement increased linearly with interlayer thickness, and the most unfavorable zones shifted from the pile-top soil to the soft soil–structure interface. This highlights how soft soil interlayers weaken the anchoring effect of the sheet pile and exacerbate interface slip and the accumulation of plastic deformation.

This study demonstrates that the failure mode of the support system is influenced by the combined effects of bolt confinement strength, prestress regulation efficiency and geological non-uniformity. For deep excavation projects, it is recommended to adopt a layered anchoring strategy to suppress the development of deep slip surfaces. The integration of prestress technology should be employed to optimize the initial stress field and reduce the risk of local deformation. In strata with weak interlayers, measures to enhance interface shear resistance or the use of composite reinforcement schemes are advised. The findings of this research provide theoretical support for stiffness matching design, critical risk zone prediction, and the stability enhancement of support structures in complex geological conditions, while emphasizing the importance of dynamic construction monitoring and adaptive design for geological conditions.

## 6. Conclusion

Based on centrifuge tests and the use of digital image measurement technology, this study focused on the variation of excavation displacement, soil pressure, the axial force of the tie-rod, and the soil displacement field under different working conditions, in order to reveal the reinforcement mechanism of a new multi-anchor sheet pile used to reinforce the bank wall of a harbor basin. The main conclusions obtained are as follows:

(1) Excavation depth is the dominant factor affecting the mechanical response (sensitivity coefficient 2.3). In contrast, tie-rod horizontal spacing shows low sensitivity (0.018), suggesting that spacing can be optimized for cost-effectiveness without significantly compromising stability.
(2) The multi-anchor system effectively restructures stress transmission. The strategic installation of anchor rods suppresses shallow soil shear deformation and shifts the potential slip surface toward the reinforced zone, enhancing overall stability.
(3) Soft soil interlayers significantly degrade system performance by inducing load superposition and extending the sliding surface. Thicker interlayers lead to nonlinear increases in horizontal earth pressure and a reduction in anchor uplift resistance.
(4) The composite system, driven by the synergistic interaction of tie-bars and prestressing, achieves a 71.9%–77.3% reduction in sheet pile bending moments compared to conventional methods. This stiffness-stress coupling effect validates the system’s suitability for deep foundation pits.

## Supporting information

S1 Data The Minimal Data Set. (XLSX)

## Abbreviations

APRR: Insertion ratio of anchor plate
APRSR: Stiffness ratio of anchorage plate
APS: The prestressed of anchor
BMssp: Bending moment of steel sheet pile
DDD: Excavation depth of harbor basin
HASP: Horizontal gap of anchor pile
Hdisp: Horizontal displacement
HSTR: Horizontal spacing of tie rods
PIV: Particle image velocimetry
SF: Safety factor
SSPPR: Penetration ratio of steel sheet pile
SSPS: Steel sheet pile stiffness
TRSR: Stiffness ratio of tie rods
TSSL: Soft soil interlayer thickness
Vdisp: Vertical displacement.
